# Demoralization syndrome among women exposed to war: comparing breast cancer survivors and those with no breast cancer history

**DOI:** 10.3389/fpsyg.2026.1812342

**Published:** 2026-08-14

**Authors:** Svetlana Baziliansky, Wafaa Sowan

**Affiliations:** University of Haifa, School of Social Work, Haifa, Israel

**Keywords:** age-related differences, breast cancer survivors, cancer survivorship, demoralization syndrome, psychological vulnerability, war exposure

## Abstract

**Introduction:**

Demoralization syndrome, characterized by distress and subjective incompetence, is prevalent among cancer survivors and particularly salient among breast cancer survivors (BCS). Collective trauma may exacerbate existential vulnerability and heighten demoralization syndrome. However, no study has compared demoralization syndrome among BCS and non-BCS women exposed to the same war-related context, nor examined age as a moderator of this relationship. Therefore, this study aimed to: (a) compare levels and clinically significant prevalence of demoralization syndrome between BCS and non-BCS women exposed to the October 7 war; (b) examine the association between war exposure and demoralization syndrome in both groups; and (c) test whether this association was moderated by breast cancer survivorship status and age.

**Materials and methods:**

This secondary analysis combined data from two parallel Israeli studies conducted during April–July 2024, following the October 7 war. The sample comprised 350 women: 190 BCS (diagnosed 1–2 years prior, post–active treatment) and 160 non-BCS participants. Measures included the War Exposure Index and Demoralization Scale-II. Group differences were examined using *t*-tests and chi-square analyses. Moderation analyses (PROCESS Model 1, 5,000 bootstrap samples) tested whether group and age moderated the association between war exposure and demoralization syndrome, controlling for age and ethnicity where appropriate.

**Results:**

BCS reported significantly higher demoralization than non-BCS participants, with 47.9% meeting criteria for demoralization syndrome versus 33.1% of non-BCS women. War exposure was positively associated with demoralization syndrome in both groups, but this association was significantly stronger among BCS. Moderation analyses indicated that war exposure predicted demoralization only among BCS. Age moderated this association in BCS: war exposure and demoralization syndrome were significantly associated among median and older participants, but not among younger women. Johnson–Neyman analysis identified age 46.22 years as the threshold above which war exposure was significantly associated with higher demoralization syndrome.

**Discussion:**

Findings suggest compounded vulnerability among BCS exposed to war stress, consistent with cumulative stress and resource-depletion frameworks. Older BCS appear particularly susceptible under dual health and societal threat. These results underscore targeted screening and psychosocial interventions addressing loss of meaning, helplessness, and existential distress among cancer survivors during armed conflict.

## Introduction

The reported prevalence of demoralization syndrome among cancer survivors ranges from 13.5 to 49.4% (Wang et al., 2023). Demoralization syndrome is defined by two core components: distress and subjective incompetence. These components manifest as loss of meaning and purpose in life, powerlessness, hopelessness, dysphoria, and perceived failure (Kissane et al., 2004; Wang et al., 2023). Kissane et al. (2004) reported that 30–40% of women with breast cancer experience clinically significant demoralization in the posttreatment period, especially those coping with ongoing uncertainty about recurrence. Demoralization syndrome is particularly salient among BCS and may partly reflect breast cancer-specific stressors, including disruptions to identity, bodily integrity, menopausal symptoms, femininity, sexual desirability, and future orientation (Sender et al., 2025; Xiong et al., 2025). Beyond these illness-specific stressors, demoralization among BCS may be understood through a liminality framework: although women are often expected to resume normal life after completion of active treatment, survivorship may remain marked by ongoing surveillance, fear of recurrence, persistent treatment-related symptoms, and for many women, prolonged endocrine therapy (Blows et al., 2012; Ganz et al., 2013; Vehling et al., 2017; Wilson, 2019). This discrepancy between social and personal expectations of recovery and the continued experience of illness-related uncertainty may undermine perceived control, agency, and confidence in the ability to cope (Kissane et al., 2004; Wang et al., 2023). In this sense, demoralization among BCS may arise from not only disease severity but also a liminal state of being medically posttreatment while still psychologically and physically living with cancer-related threat (Blows et al., 2012; Wilson, 2019). Such a mismatch may intensify subjective incompetence, helplessness, and loss of meaning, which are central features of demoralization syndrome (Kissane et al., 2004; Wang et al., 2023).

### Demoralization syndrome and war exposure

War-related conflicts are frequently characterized by prolonged uncertainty and disruptions to social and economic stability. Such conditions undermine coping resources and intensify feelings of helplessness and loss of purpose, which constitute core elements of demoralization (Vesco et al., 2025). To the best of our knowledge, apart from two studies conducted in Israel in response to the October 7 war examining demoralization (Baziliansky and Sowan, 2025; Sowan and Baziliansky, 2024), no additional research has been identified that specifically investigated demoralization in a war context. Sowan and Baziliansky (2024) found that higher exposure to war was associated with higher levels of demoralization symptoms and posttraumatic stress symptoms. Moreover, the impact of war exposure on posttraumatic stress symptoms was contingent on demoralization levels; only at moderate and high levels of demoralization symptoms did exposure to war significantly affect posttraumatic stress symptoms. Baziliansky and Sowan (2025) revealed that the higher the indirect exposure to war, the higher the acute stress symptoms, use of disengaged coping, and demoralization. These findings suggest that war exposure can erode individuals’ sense of purpose, competence, and future orientation, thereby fostering demoralization. Such processes are likely to be intensified among individuals already contending with the profound existential threat and uncertainty posed by cancer.

### Breast cancer survivors and demoralization syndrome in a war-related context

Empirical research has indicated that war-related conflicts can amplify psychological distress, existential threat, and illness-related vulnerability (Baziliansky and Sowan, 2025; Keinan-Boker et al., 2011; Vesco et al., 2025). These factors may increase vulnerability to demoralization syndrome among BCS, who already face ongoing cancer-related uncertainty and threat.

Study findings regarding the association between age and demoralization syndrome among cancer survivors remain limited and inconsistent. A study conducted in Taiwan among older cancer survivors aged 61 years or older reported no significant relationship between age and demoralization (Ko et al., 2018). Conversely, another study demonstrated a more complex pattern: among cancer survivors receiving palliative treatment, the effect of age on demoralization syndrome was negative for women, indicating that younger women experienced higher levels of demoralization syndrome, whereas for men in palliative care, age had a positive association with demoralization (Vehling et al., 2013). Among cancer survivors receiving curative treatment, however, the effect of age on demoralization syndrome was not significant. Notably, female gender emerged as a risk factor for demoralization syndrome only among younger women undergoing palliative treatment (Vehling et al., 2013).

Building on this literature, the present study addresses two unresolved questions. First, although previous studies have established the clinical relevance of demoralization among women with breast cancer, this work has primarily examined demoralization in the illness and survivorship context (Huang et al., 2025; Kissane et al., 2004; Vehling et al., 2017). Beyond clinical cancer samples, recent research has also examined demoralization in non-BCS or non-clinical groups, showing relatively low baseline levels and providing normative values by age and sex (Ramm et al., 2025; Sender et al., 2025). However, these lines of research have not been integrated through a direct comparison of BCS and non-BCS women exposed to the same large-scale societal stressor. Such a comparison is needed to clarify whether demoralization reflects a general psychological response to collective trauma or recent breast cancer survivorship confers additional vulnerability when war-related stress occurs against the background of cancer-related uncertainty (Baziliansky and Sowan, 2025; Sowan and Baziliansky, 2024; Vesco et al., 2025). Second, the role of age in this association remains unclear, despite evidence that age may shape cancer-related coping, survivorship experiences, and vulnerability to existential distress (Ko et al., 2018; Vehling et al., 2013). The present study, therefore, extended prior work by examining demoralization among BCS and non-BCS women exposed to the October 7 war and testing whether age moderates the association between war exposure and demoralization.

### The present study

The present study was guided by the frameworks of cumulative stress, resource depletion, and liminality. Together, these frameworks suggest that recent breast cancer survivorship may constitute an ongoing context of cancer-related uncertainty, diminished perceived control, bodily and psychosocial disruption, and concern about recurrence. Therefore, when war-related stress occurs against this background, it may further deplete coping resources and intensify feelings of helplessness, subjective incompetence, and loss of meaning. Thus, rather than assuming that prior cancer survivorship would reduce vulnerability to war-related demoralization, we expected BCS to show greater vulnerability under conditions of dual health-related and societal threat.

The purpose of this study was to examine whether demoralization syndrome differs between BCS and non-BCS women exposed to the same war-related context, whether war exposure is associated with demoralization syndrome, and whether this association is moderated by breast cancer survivorship status and age. We hypothesized:

1 Demoralization syndrome would be more prevalent and more severe among BCS than among non-BCS women.
2 Higher levels of war exposure would be associated with higher levels of demoralization syndrome, and this association would be stronger among BCS than among non-BCS women.
3 Age would moderate the association between war exposure and demoralization syndrome. Given the limited and inconsistent evidence regarding age and demoralization among cancer survivors, this hypothesis was exploratory rather than a strictly directional prediction.

## Materials and methods

### Participants and procedure

This secondary analysis combined data from two longitudinal studies collected in parallel by the same research institute. The first study focused on exposure to war and its effects on the non-BCS group (Approval No. 081/24), whereas the second explored the well-being of BCS in the early posttreatment survivorship phase. Because survivorship definitions vary by cancer type, we operationally defined BCS for this study as women who had fully completed primary active medical treatments (i.e., surgery, chemotherapy, and/or radiation) within the past 6 to 24 months (Approval No. 106/24). Both studies were approved by the ethics board of the Faculty of Social Welfare and Health Sciences at the University of Haifa. To achieve heterogeneous representation, the samples included diverse social groups, encompassing a range of ages, ethnicities, and income levels. Data were collected between April and July 2024.

The non-BCS group featured 160 women. Inclusion criteria were that respondents had to be aged 20 or older and have sufficient understanding of Hebrew or Arabic to complete the survey. Potential participants were screened for eligibility; individuals reporting chronic health conditions, major psychiatric events, or cognitive decline in the 6 months prior to the war were excluded to minimize confounding from preexisting conditions.

Various steps were taken to recruit participants. First, potential participants were identified based on age and area of residence through HSR Panel, a national survey company that maintains a large, diverse panel of pre-screened participants from across Israel. Eligible panel members were then contacted by email or text message with an invitation to participate. The invitation included a brief description of the study. Those who agreed to participate provided online informed consent and received a link to the questionnaire. The survey took approximately 15–20 min to complete. Participants from the general population were compensated through the survey company’s standard panel reward system, in which respondents accumulate redeemable points through participation. The BCS group included 190 women. The inclusion criteria were receiving a diagnosis of Stage 0–4 breast cancer^1^ 1–2 years prior to enrollment in the study, completion of active treatment, and sufficient knowledge of either Hebrew or Arabic. Participants were recruited from the same survey company, whose database contained 18,000 BCS (diagnoses were verified by medical documentation). Approximately 1,500 had been diagnosed in the last 1–2 years. Approximately 1,000 of these BCS were purposively sampled to represent a wide age range, including Jewish and Arab women, varying levels of secularism and religiosity, and diverse residential areas. The number of Arab participants was increased beyond their share of the population to ensure a sufficient subsample for analysis. The first 190 women who agreed to participate were enrolled in the study and signed an informed consent form. Although both groups were recruited through the same survey panel, participants in the BCS group received a direct incentive of approximately US$13 (45 new Israeli shekels) because they represented a targeted population requiring specific recruitment. In contrast, participants from the general population were compensated through the survey company’s standard panel reward system, in which respondents accumulated points that could later be redeemed for rewards.

Based on *a priori* power calculation using G*Power for multiple linear regression (with 20 independent variables), a sample size of 157 participants was required to achieve statistical power of 0.80 at an alpha level of 0.05. To increase statistical precision and ensure sufficient power to detect interaction effects in the moderation analyses, a larger sample of 350 participants was targeted for recruitment. Recruitment was terminated upon reaching this final sample size, with the group breakdown being 190 participants in one group and 160 in the other.

Table 1 presents the sociodemographic characteristics of both groups. Overall, the two groups were similar in most background characteristics. Both predominantly included women in middle adulthood and had comparable religiosity patterns. In both samples, most women were married or in a partnership and had, on average, one or two children.

**Table 1:** Sociodemographic and health characteristics of the study participants (*N* = 350).

Variable	Non-BCS (*n* = 160)		BCS (*n* = 190)		*t* or *χ*^2^
	*n* (%) *or M* (SD)	Range	*n* (%) *or M* (SD)	Range	
Age, years	46.6 (11.17)	25–79	51.57 (16.52)	21–81	*t*_(348)_ = −5.76*
Education, years	13.89 (2.96)	3–23	14.81 (3.12)	2–25	*t*_(348)_ = −2.79*
Number of children	1.53 (1.74)	0–11	1.72 (1.31)	0–6	*t*_(348)_ = −1.17
Ethnicity					
Arab	84 (52.5)		54 (28.4)		*χ*^2^_(1)_ = 15.69*
Jewish	76 (47.5)		136 (71.6)		
Marital status					
Single	61 (38.1)		47 (24.7)		*χ*^2^_(4)_ = 12.87*
Married or partnered	86 (53.8)		118 (62.1)		
Divorced	12 (7.5)		14 (7.9)		
Widowed	1 (0.6)		10 (5.3)		
Employment					
Employed	81 (50.6)		125 (65.8)		*χ*^2^_(3)_ = 11.45*
Unemployed	45 (28.1)		29 (15.3)		
Retiree	34 (21.3)		36 (18.9)		
Cancer stage at diagnosis					
Stage 0			15 (7.9)		
Stage 1			36 (18.9)		
Stage 2			73(38.4)		
Stage 3			38(20.0)		
Stage 4			28 (14.7)		

BCS = breast cancer survivors; M = mean; SD = standard deviation. *p* < 0.01.

However, several differences emerged. Women in the BCS group were slightly older on average (51.57 vs. 46.6 years) and reported somewhat higher educational attainment. The BCS group also had a higher proportion of Jewish participants (71.6% vs. 47.5%) and a higher employment rate (65.8% vs. 50.6%). These distinctions provide context for interpreting the subsequent analyses.

Regarding cancer-related variables, the subsample included participants diagnosed at all stages of breast cancer, with the largest percentage being Stage 2. The types of medical treatment were as follows: 83.2% underwent surgery, 46.8% received radiotherapy, and most participants also received chemotherapy, immunotherapy, or targeted therapy.

### Measures

Background details included respondents’ age, family status, education level, health status, economic status, religiosity, number of children, area of residence, ethnicity, and illness-related characteristics.

The War Exposure Index was modified from previous research on the Second Lebanon War (Cohen, 2008; Yahav and Cohen, 2007). This index demonstrated good reliability and validity in earlier research on the October 7 war (Baziliansky and Sowan, 2025; Sowan and Baziliansky, 2024). The index assessed respondents’ exposure to war events. The index features eight items: whether acquaintances or relatives have been injured, killed, or kidnapped; subjective feelings of danger; property or home damage; employment or income damage; and intensity of watching TV coverage (including live news, extensive reports on the war and casualties, and discussions and analysis by experts). Each item, except the first, was measured on a 4-point Likert scale (0 = *not at all*, 3 = *very much*). We created a dichotomous variable by recoding 0 or 1 as 0 (meaning lower intensity) and 2 or 3 as 1 (indicating higher intensity). An example question is: “Has your income or employment been negatively affected since the war broke out?” Only the first question related to acquaintances or relatives who were harmed, and respondents were asked to report “yes” or “no.” Based on the sum of responses, a total score was produced that ranged from 0 to 8 (Bromet et al., 2016).

The Demoralization Scale II is the short version of the Demoralization Scale-24, the most widely validated instrument for assessing demoralization among patients with chronic health conditions (Clarke and Kissane, 2002). The 3-point self-report scale ranges from 0 (*never*) to 2 (*often*) and features 16 items across two subscales (meaning and purpose, distress and coping ability). Given its revalidation, psychometric strengthening, and simplification, the scale is an improved and more practical measure of demoralization for research and clinical use (Robinson et al., 2016a, 2016b). Example statements are: “I do not cope well with life” and “I feel that I cannot help myself.”

To determine clinically significant levels of demoralization, we adopted Robinson et al.’s (2016a, 2016b) suggested method of categorizing the total score into three levels: low (0–3), moderate (4–10), and severe (≥11) demoralization. This scale has been used in previous studies and was shown to be reliable (*α* = 0.86–0.92; Liao et al., 2018; Wu et al., 2019). The questionnaire was translated into Hebrew and Arabic, and its internal reliability coefficients in a previous study were found to be acceptable (Sowan and Kissane, 2024). The internal consistency in the present study (Cronbach’s alpha) was *α* = 0.93.

### Data analysis

Statistical analyses were performed using IBM SPSS Statistics version 28.0. Descriptive statistics were calculated to summarize the study variables, followed by Pearson correlation to examine bivariate relationships. To test whether the magnitude of these correlations differed significantly between the two groups, Fisher’s *z*-tests were utilized. Independent-samples *t*-tests were conducted to compare mean differences in war exposure and demoralization between the BCS and non-BCS groups. A chi-square test of independence was used to compare the prevalence of demoralization between the BCS and non-BCS groups. All primary analyses were conducted while statistically controlling for age and ethnicity, because both were significantly associated with the study variables.

In addition, two moderation analyses were conducted using the PROCESS macro Model 1 (Hayes, 2017) with 5,000 bootstrap samples to generate 95% confidence intervals. Continuous predictor variables (exposure to war and age) were mean-centered prior to running the regression models. The first model tested whether the population group moderated the relationship between war exposure and demoralization, and the second model tested whether age moderated that relationship (analyzed separately for both the BCS and non-BCS groups). The moderation analysis for age was conducted by probing conditional effects at three points based on age percentiles: 16th percentile (younger), 50th percentile (median), and 84th percentile (older). Additionally, the Johnson–Neyman technique was applied to identify the specific range of values for the moderator (age) for which the interaction effect was significant. We performed a Johnson–Neyman analysis to determine whether the effect of war exposure on demoralization syndrome varied in significance as a function of age. A threshold of *p* < 0.05 was used to determine statistical significance for all analyses.

## Results

### Descriptive statistics and bivariate correlations

The means, standard deviations, ranges, and correlations of the study variables are presented in Table 2. As shown in the table, in the non-BCS group, the only significant correlation occurred between war exposure and demoralization. In the BCS group, war exposure was also positively associated with demoralization, indicating that greater exposure was associated with higher levels of demoralization syndrome. A test comparing these correlations confirmed that the association was significantly stronger in the BCS group than in the non-BCS group (*z* = −2.61, *p* = 0.005). Conversely, age was negatively associated with demoralization syndrome, suggesting that older participants reported lower demoralization.

**Table 2:** Statistics and correlations of study variables for BCS and non-BCS.

Variable	Non-BCS (*n* = 160)			BCS (*n* = 190)		
	*M* (SD)	1	2	*M* (SD)	1	2
1. Demoralization	7.37 (7.15)			10.50 (7.19)		
2. War exposure	16.73 (2.42)	0.17*		16.14 (2.66)	0.40**	
3. Age	46.6 (11.17)	−0.09	−0.04	51.57 (16.52)	−0.20*	−0.03

BCS = breast cancer survivors; M = mean; SD = standard deviation. *p < 0.05, **p < 0.001.

### Group comparisons: war exposure and demoralization (hypothesis 1)

All subsequent analyses were conducted while statistically controlling for age and ethnicity, because both were associated with the study variables. An independent-samples *t*-test revealed a significant difference in exposure to war between the two groups, *t*_(348)_ = 2.15, *p* = 0.032. Participants in the non-BCS group reported significantly higher exposure levels compared to those in the BCS group. Additionally, a second *t*-test revealed that BCS reported significantly higher levels of demoralization compared to the non-BCS group, *t*_(348)_ = −4.06, *p* < 0.001. Additionally, in the non-BCS group, 39% had no demoralization, 27.5% had moderate demoralization, and 33.1% met criteria for demoralization syndrome. Among BCS, 17.4% had no demoralization, 34.6% had moderate levels, and 47.9% met criteria for demoralization syndrome. The results indicate a significant difference between the two samples, *χ*^2^_(1, 350)_ = 7.83, *p* = 0.005. BCS were more likely to meet criteria for demoralization syndrome (47.9%) compared to non-BCS (33.1%). As hypothesized, demoralization syndrome was more prevalent among BCS than in the non-BCS group, supporting our first hypothesis.

### The moderating role of population group (hypothesis 2)

To examine the second hypothesis, a moderated regression analysis (PROCESS Model 1) was performed with group (non-BCS vs. BCS) as the moderator of the relationship between exposure to war and demoralization (see Table 3). Age and ethnicity were entered as covariates. The continuous predictors (exposure to war and age) were mean-centered prior to analysis. The overall model was statistically significant (*F*_(3, 346)_ = 17.76, *p* < 0.001), explaining 14% of the variance in demoralization. A significant moderating effect of group (non-BCS vs. BCS) on the relationship between exposure to war and demoralization was found (*b* = 0.67, *t* = 2.31, *SE* = 0.29, *p* = 0.021). The interaction term contributed a significant 4% to the explained variance in demoralization. The main effects of exposure to war (*b* = −0.28, *p* = 0.558) and group (*b* = −7.52, *p* = 0.124) were found to be not statistically significant in this model.

**Table 3:** Conditional effects of exposure to war on demoralization.

Moderator	*b*	*SE*	Bootstrap 95% CI	*p*
Non-BCS	0.38	0.22	−0.05, 0.83	0.091
BCS	1.06	0.18	0.71, 1.41	< 0.001

*b* = unstandardized regression coefficient; SE = standard error; CI = confidence interval.

**Figure 1:**
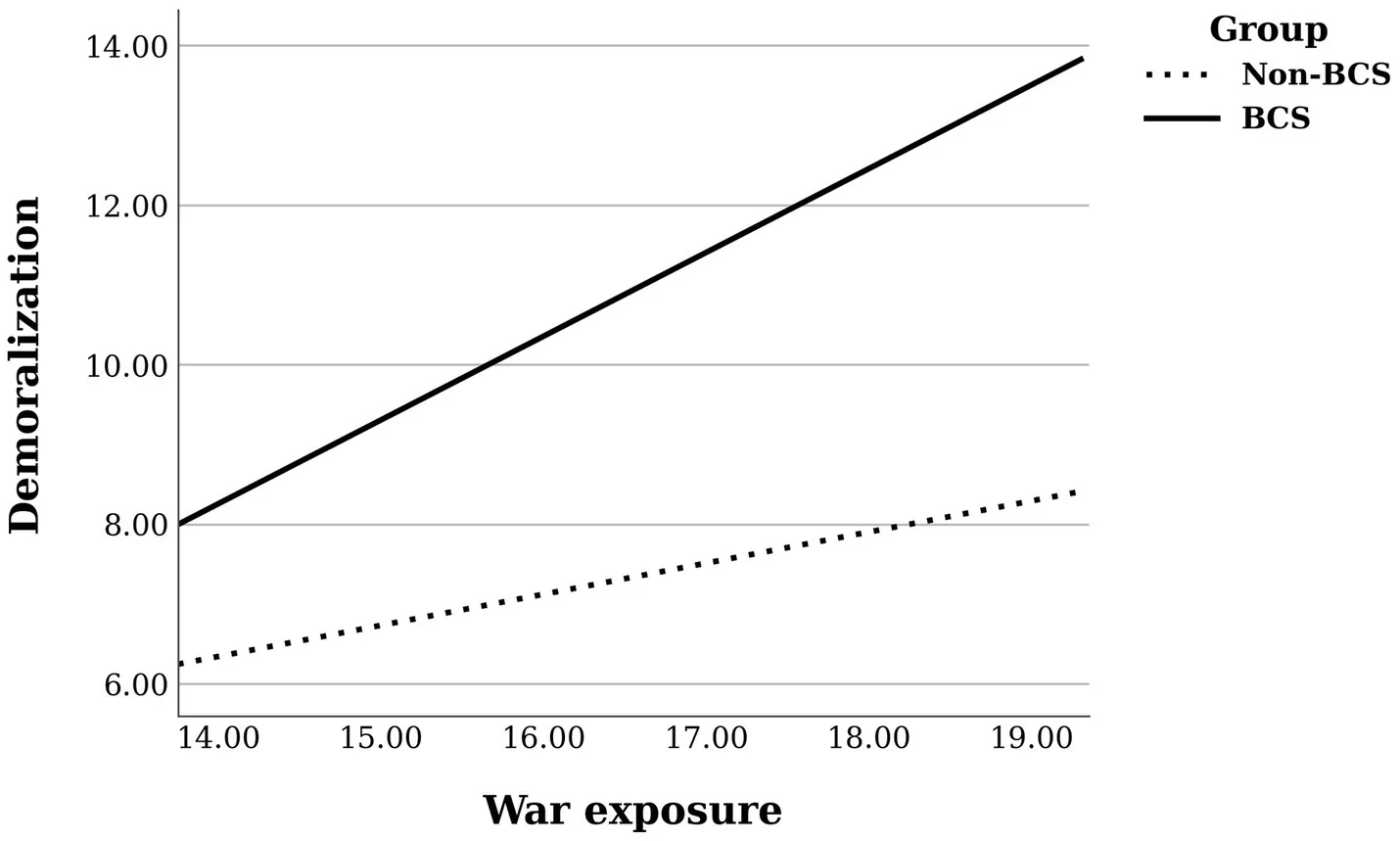
Results of the moderation model of exposure to war on demoralization in non-BCS and BCS.

A graphic representation of the analysis (Figure 1) visually demonstrates these contrasting slopes, wherein the steep solid line represents the BCS group and the lower, dotted line represents the non-BCS group. The association between exposure to war and demoralization was steep, positive, and statistically significant for the BCS group (*b* = 1.06, *t* = 5.67, *p* < 0.001). In contrast, this trajectory was relatively flat and not statistically significant for the non-BCS group (*b* = 0.38, *t* = 1.73, *p* = 0.091). In practical terms, this indicates that higher exposure to war was linked to a sharp increase in demoralization only among breast cancer survivors, whereas the non-BCS group’s demoralization levels remained relatively stable regardless of war exposure. This means that the association between exposure to war and demoralization was stronger among BCS, providing support for our second hypothesis.

### The moderating role of age (hypothesis 3)

The third hypothesis, which involved age as a moderator of the relationship between exposure to war and demoralization, was tested separately for the two study groups (BCS and non-BCS) using PROCESS Model 1. In this analysis, prior to running the analysis, the continuous predictor (exposure to war) and the moderator (age) were mean-centered.

The moderation analysis for the non-BCS group indicated that the overall model was not significant (*F*_(3, 156)_ = 1.46, *p* = 0.228, *b* = 0.15, *t* = 0.96, *SE* = 0.16, *p* = 0.339). This indicates that in the non-BCS group, age did not moderate the relationship between war exposure and demoralization. However, in the BCS group, the moderation analysis was significant (*F*_(3,186)_ = 6.08, *p* = 0.02), explaining 13.4% of the variance in demoralization. The interaction added 3% to the explained variance of demoralization.

**Figure 2:**
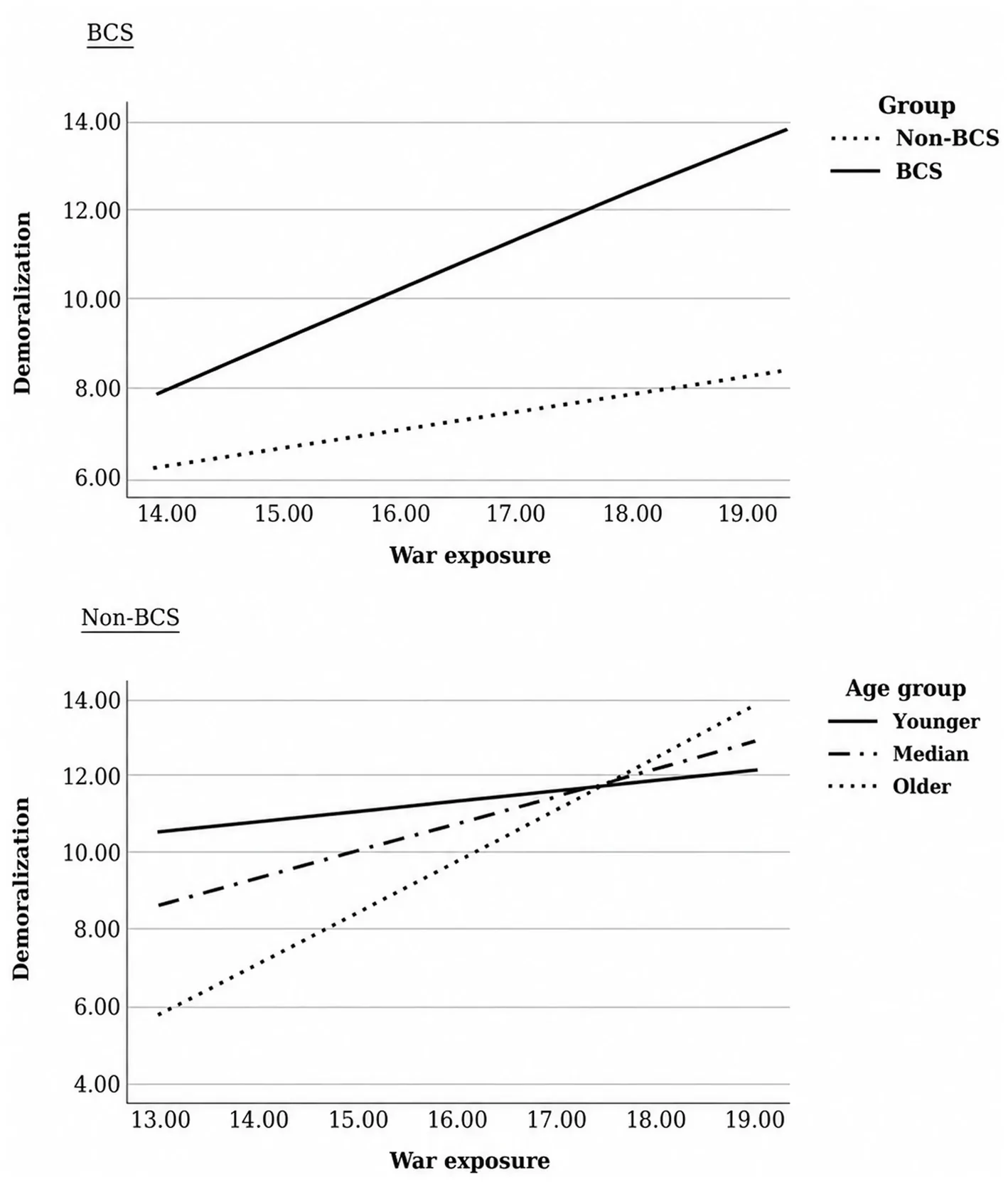
Results of the moderation model of exposure to war on demoralization in the BCS group across age groups.

To interpret this interaction, we examined the conditional effects of war exposure on demoralization at three age levels: younger (16th percentile), median (50th percentile), and older (84th percentile). As detailed in Table 4 and illustrated by the simple slopes in Figure 2, the interaction was characterized by three distinct visual trajectories mapped across different line types. The slope of the relationship between exposure to war and demoralization was relatively flat and not statistically significant in the younger group (represented by the solid line; *b* = 0.26, *SE* = 0.30, *p* = 0.387). However, the trajectory shifted to become positive and statistically significant for the median age group (represented by the dash-dot line; *b* = 0.70, *SE* = 0.20, *p* < 0.001) and was steepest for the older group (represented by the dotted line; *b* = 1.32, *SE* = 0.30, *p* < 0.001). These results indicate that age moderated the positive relationship between exposure to war and demoralization in the BCS group. Specifically, as age increased, the positive association between war exposure and demoralization became stronger.

**Table 4:** Conditional effects of exposure to war on demoralization for BCS.

Moderator	Condition	*b*	SE	Bootstrap 95% CI	*p*
Age (−1 SD)	Younger	0.26	0.30	−0.33, 0.86	0.38
Age (1 SD)	Median	0.70	0.19	0.31, 1.09	<0.001
Age (+1 SD)	Older	1.32	0.30	0.73, 1.91	<0.001

BCS = breast cancer survivors; *b* = unstandardized regression coefficient; SE = standard error; CI = confidence interval.

Therefore, our third hypothesis was partially supported: Moderation was found only in the BCS group, such that older age was associated with a stronger impact of war exposure on demoralization. The Johnson–Neyman analyses further clarified age-related differences by identifying a threshold at 46.22 years. Specifically, the detrimental effects of war exposure became statistically significant only among individuals above this age, indicating that war exposure is associated with poorer outcomes beyond this point.

## Discussion

The present study examined demoralization among BCS and non-BCS groups in the context of war exposure, offering a new contribution to the literature by directly comparing these two groups exposed to the same warfare trauma. Consistent with our first hypothesis and in line with prior research identifying cancer survivors as especially vulnerable to existential distress (Huang et al., 2025; Vehling et al., 2017), BCS in this study reported significantly higher levels of demoralization compared to the non-BCS group. Nearly half of the BCS sample met criteria for demoralization syndrome, underscoring the profound and lasting biopsychosocial burden faced by this group, even after treatment completion (Ganz et al., 2013), which may intensify in periods of external threat (Sowan and Baziliansky, 2024; Vesco et al., 2025).

The October 7 war represents an additional, concurrent stressor that can compound illness-related uncertainty by introducing sustained insecurity and disruptions to routines and access to services. These conditions may erode perceived control and existential coherence, thereby heightening the risk of demoralization syndrome among cancer survivors in a wartime context (Ben-Arye et al., 2025; Raut, 2025). Notably, similar patterns were observed during the COVID-19 pandemic, supporting our findings. Pandemic-related stressors and disruptions to oncological care were associated with elevated anxiety, depressive symptoms, posttraumatic stress, and fear of cancer recurrence among cancer survivors (Doege et al., 2025; Marino et al., 2022; Massicotte et al., 2021). These outcomes map onto core features of demoralization—helplessness, hopelessness, and diminished meaning (Kissane et al., 2001; Tecuta et al., 2015)—suggesting that the cumulative impact of cancer- and pandemic-related stressors may increase susceptibility to demoralization syndrome among cancer survivors. Taken together, the present findings may reflect a “double jeopardy” process in which chronic cancer-related threat is compounded by acute and ongoing war-related stress, resulting in heightened demoralization in the BCS group.

A central contribution of this study lies in demonstrating that war exposure predicted demoralization in both groups, yet exerted a significantly stronger effect among BCS (second hypothesis). This interaction pattern suggests that cancer-related existential vulnerability may heighten sensitivity to additional stressors such as war. The amplification of demoralization under dual stress exposure aligns with cumulative stress and resource-depletion frameworks, whereby repeated or layered threats erode coping capacity and increase susceptibility to meaning disruption (Hobfoll, 1989; McEwen, 1998). Individuals already grappling with intrusive bodily reminders of cancer and uncertainty about the future may be particularly ill-equipped to manage an overwhelming traumatic experience such as the October 7 war, which involved a serious threat to the security and physical integrity of the civilian population (Palgi et al., 2024). Consistent with this interpretation, studies conducted following the outbreak of the October 7 war have demonstrated high levels of acute stress reactions (Baziliansky and Sowan, 2025) and posttraumatic stress (Sowan and Baziliansky, 2024) among Israeli civilians nationwide. Greater exposure to war-related events predicted more severe psychological symptoms (Gilboa Schechtman et al., 2025; Levi-Belz et al., 2025). Importantly, both acute and posttraumatic stress were linked to processes reflecting loss of meaning and depleted coping, including heightened event centrality and reliance on disengaged coping strategies (Baziliansky and Sowan, 2025; Gilboa Schechtman et al., 2025; Levi-Belz et al., 2025).

Another important finding relates to the moderating role of age. Moderation was found only in the BCS group. Specifically, in the median and older age groups, the association between war exposure and demoralization was stronger, whereas no significant association was found in the younger group. These findings partially support our third hypothesis and contribute to the inconsistent literature regarding age and demoralization in cancer contexts (Ko et al., 2018; Vehling et al., 2013).

Several interpretations are possible. Older women may experience cumulative health-related stressors and caregiving demands, increasing their vulnerability to additional external threats (Rodin et al., 2009; Hobfoll, 1989). Alternatively, younger survivors may experience a distinct psychological profile characterized by greater flexibility, future-oriented coping, and access to digital-social support systems, which may buffer existential distress during traumatic events, such as war (Carver and Connor-Smith, 2010; Bellizzi et al., 2012).

The present findings also have significant methodological and conceptual implications for the study of demoralization syndrome. First, significant differences in exposure to the October 7 war between groups, with the non-BCS group reporting slightly higher exposure yet lower demoralization syndrome, emphasize that exposure severity alone does not account for psychological impact. Rather, individual vulnerability factors, including cumulative health stressors, existential insecurity, and diminished perceived agency, may heighten sensitivity to additional stress exposure and increase the risk of demoralization (Hobfoll, 1989; Kissane et al., 2001; McEwen, 1998). These frameworks posit that when individuals experience cumulative or chronic stress, even moderate additional adversity may exceed their coping resources and precipitate meaning collapse.

Second, the high prevalence of clinically significant demoralization in both groups suggests that war-related contexts may generate population-wide psychosocial risk, extending beyond those with preexisting health conditions. Research on collective trauma indicates that large-scale societal stressors can disrupt psychological functioning and strain resilience across broad segments of the population (Bonanno et al., 2011; Galea et al., 2020). In this context, exposure to warfare may operate as a destabilizing force that weakens coping resources across diverse subgroups, with more pronounced effects among medically vulnerable populations such as BCS.

This study has several limitations that warrant consideration. The reliance on self-report measures may have introduced response biases. Although the use of validated tools such as the Demoralization Scale II strengthened the assessment, clinical interviews would offer a more comprehensive evaluation. The study also did not assess additional psychological variables that may influence demoralization syndrome, such as prior psychological distress, anxiety and depressive symptoms, fear of cancer recurrence, coping strategies, perceived social support, resilience, and prior trauma exposure. These variables may contribute to individual differences in demoralization and may partly account for the observed associations between war exposure, breast cancer survivorship status, age, and demoralization syndrome. Moderation analyses provided insight into statistical interaction effects; however, future research using repeated measures across different war phases, as well as pre-war or pre-diagnosis baseline assessments where possible, would allow stronger conclusions regarding temporal change and directionality. Furthermore, despite efforts to recruit diverse samples, online survey methods may underrepresent individuals with limited digital access or lower socioeconomic resources. In addition, although both study groups received incentives through the survey company, the form of compensation differed between groups: BCS received direct monetary payment, whereas participants from the general population received redeemable panel points. This difference may have influenced participation patterns and should be considered when interpreting the findings. Finally, the study focused exclusively on women, aligning with the clinical relevance of breast cancer but limiting generalizability to men or nonbinary populations.

Despite these limitations, the study offers a new understanding of demoralization syndrome in both cancer survivorship and trauma- and war-exposed civilian populations. The findings underscore the compounded psychological burden experienced by BCS during the October 7 war and highlight age as an important contextual factor shaping vulnerability. Clinically, these results suggest the need for targeted screening and psychosocial support for demoralization syndrome among BCS during warfare, with particular attention to older survivors. Interventions aimed at restoring meaning, strengthening coping capacities, and addressing existential concerns may be especially beneficial. Given that demoralization syndrome has been linked to reduced treatment adherence, poorer quality of life, and heightened psychological distress (Nanni et al., 2018), addressing this construct is essential not only in oncology care but also in emergency and community mental health settings.

Future research should explore protective factors that may buffer demoralization in high-risk groups of cancer survivors, such as social support (Tang et al., 2025), hope (Nikoloudi et al., 2025), and self-compassion (Bitan et al., 2025), which, to the best of our knowledge, have yet to be examined in relation to demoralization. Researchers should examine whether interventions targeting existential resilience can mitigate the compounding effects of health and societal stressors. Longitudinal designs spanning prewar, active conflict, and postwar phases would further elucidate trajectories of demoralization and inform the timing of intervention delivery.

To conclude, the present study provided new, compelling evidence that war exposure disproportionately elevates demoralization among BCS compared to the non-BCS group, particularly among older survivors. These findings highlight the need for integrative psychosocial care models that acknowledge the interplay between medical vulnerability and broader sociopolitical contexts, ensuring that the psychological well-being of cancer survivors is safeguarded even amid traumatic societal conditions such as war.

## Data Availability

The raw data supporting the conclusions of this article will be made available by the authors, without undue reservation.

## Footnotes

1 Tumor staging describes the size and extent of cancer spread in the body. In this study, staging was determined using the TNM classification system, which evaluates three main factors: size and extent of the primary tumor, number of nearby lymph nodes that have cancer, and whether the cancer has metastasized, or spread, to other organs. Stages range from 0 to 4, with higher numbers indicating more advanced disease.

## References

[ref1] Baziliansky S. Sowan W. (2025). Exposure to warfare and demoralization: acute stress symptoms and disengaged coping as mediators. Eur. J. Psychotraumatol. 16:2449308. doi: 10.1080/20008066.2024.2449308, 39801397 PMC11731291

[ref2] Bellizzi K. M. Smith A. Schmidt S. Keegan T. H. M. Zebrack B. Lynch C. F. . (2012). Positive and negative psychosocial impact of being diagnosed with cancer as an adolescent or young adult. Cancer 118, 5155–5162. doi: 10.1002/cncr.27512, 22415815

[ref3] Ben-Arye E. Gressel O. Keshet Y. Vagedes J. Samuels N. Schachter V. . (2025). Chemotherapy under fire: narratives of patients with cancer during wartime. Psychooncology 34:e70327. doi: 10.1002/pon.70327, 41192952 PMC12588825

[ref4] Bitan S. Hasson-Ohayon I. Lavidor M. Daches S. (2025). The relationship between self-compassion and psychological distress in cancer patients and survivors: a systematic review and meta-analysis. J. Psychosoc. Oncol. 44, 409–427. doi: 10.1080/07347332.2025.2570787, 41082393

[ref5] Blows E. Bird L. Seymour J. Cox K. (2012). Liminality as a framework for understanding the experience of cancer survivorship: a literature review. J. Adv. Nurs. 68, 2155–2164. doi: 10.1111/j.1365-2648.2012.05995.x, 22458303

[ref6] Bonanno G. A. Westphal M. Mancini A. D. (2011). Resilience to loss and potential trauma. Annu. Rev. Clin. Psychol. 7, 511–535. doi: 10.1146/annurev-clinpsy-032210-104526, 21091190

[ref7] Bromet E. J. Hobbs M. J. Clouston S. A. Gonzalez A. Kotov R. Luft B. J. (2016). DSM-IV post-traumatic stress disorder among world trade center responders 11–13 years after the disaster of 11 September 2001 (9/11). Psychol. Med. 46, 771–783. doi: 10.1017/S0033291715002184, 26603700 PMC4754831

[ref8] Carver C. S. Connor-Smith J. (2010). Personality and coping. Annu. Rev. Psychol. 61, 679–704. doi: 10.1146/annurev.psych.093008.100352, 19572784

[ref9002] Cohen M. (2008). Acute stress disorder in older, middle-aged and younger adults in reaction to the Second Lebanon War. Int. J. Geriatr. Psychiatry 23, 34–40. doi: 10.1002/gps.1832 17503541

[ref9] Clarke D. M. Kissane D. W. (2002). Demoralization: its phenomenology and importance. Aust. N. Z. J. Psychiatry 36, 733–742. doi: 10.1046/j.1440-1614.2002.01086.x, 12406115

[ref10] Doege D. Frick J. Eckford R. D. Koch-Gallenkamp L. Schlander M. Baden-Württemberg Cancer Registry . (2025). Anxiety and depression in cancer patients and survivors in the context of restrictions in contact and oncological care during the COVID-19 pandemic. Int. J. Cancer 156, 711–722. doi: 10.1002/ijc.35204 39361297 PMC11661517

[ref11] Galea S. Merchant R. M. Lurie N. (2020). The mental health consequences of COVID-19 and physical distancing: the need for prevention and early intervention. JAMA Intern. Med. 180, 817–818. doi: 10.1001/jamainternmed.2020.1562, 32275292

[ref9001] Ganz P. A. Yip C. H. Gralow J. R. Distelhorst S. R. Albain K. S. Andersen B. L. (2013). Supportive care after curative treatment for breast cancer (survivorship care): resource allocations in low- and middle-income countries. A Breast Health Global Initiative 2013 consensus statement. Breast 22, 606–615. doi: 10.1016/j.breast.2013.07.049 24007941

[ref12] Gilboa Schechtman E. Hay D. E. Schwartz I. Neria Y. Roe D. (2025). The unfolding of psychological distress following the October 7 attack on Israel: the impact of exposure, gender, and event centrality. Psychiatry Res. 344:116356. doi: 10.1016/j.psychres.2025.116356 39798485

[ref13] Hayes A. F. (2017). Introduction to Mediation, Moderation, and Conditional Process Analysis: A Regression-Based Approach. New York, NY: Guilford Press.

[ref14] Hobfoll S. E. (1989). Conservation of resources: a new attempt at conceptualizing stress. Am. Psychol. 44, 513–524. doi: 10.1037/0003-066X.44.3.513, 2648906

[ref15] Huang Y. Zhuang P. Guan A. Ren X. Xu L. (2025). Factors influencing the demoralisation syndrome of post-operative patients with breast cancer: a cross-sectional study. Nurs. Open 12:e70130. doi: 10.1002/nop2.70130, 39731467 PMC11681367

[ref16] Keinan-Boker L. Vin-Raviv N. Liphshitz I. Linn S. Barchana M. (2011). Cancer incidence in Israeli civilians during periods of war and terror. Eur. J. Cancer 47, 2290–2297. doi: 10.1016/j.ejca.2011.04.002

[ref17] Kissane D. W. Clarke D. M. Street A. F. (2001). Demoralization syndrome—a relevant psychiatric diagnosis for palliative care. J. Palliat. Care 17, 12–21. doi: 10.1177/082585970101700103, 11324179

[ref18] Kissane D. W. Wein S. Love A. Lee X. Q. Kee P. L. Clarke D. M. (2004). The demoralization scale: a report of its development and preliminary validation. J. Palliat. Care 20, 269–276. doi: 10.1177/082585970402000402, 15690829

[ref19] Ko K.-T. Lin C.-J. Pi S.-H. Li Y.-C. Fang C.-K. (2018). Demoralization syndrome among elderly patients with cancer disease. Int. J. Gerontol. 12, 12–16. doi: 10.1016/j.ijge.2018.01.001

[ref20] Levi-Belz Y. Amsalem D. Groweiss Y. Blank C. Neria Y. (2025). The attack is not over yet: the impact of direct exposure to the October 7, 2023, attack on trajectories of PTSD and depression among the Israeli population. Psychol. Trauma 17, 1505–1513. doi: 10.1037/tra0001933, 40338540

[ref21] Liao H.-Y. Chiu C.-C. Ko Y.-Y. Chen H.-M. (2018). Factors associated with demoralisation syndrome in patients before and after cardiac surgery. J. Clin. Nurs. 27, e559–e568. doi: 10.1111/jocn.14094, 28960534

[ref22] Marino P. Touzani R. Pakradouni J. Ben Soussan P. Gravis G. (2022). The psychological distress of cancer patients following the COVID-19 pandemic first lockdown: results from a large French survey. Cancers (Basel) 14:1794. doi: 10.3390/cancers14071794, 35406566 PMC8997456

[ref23] Massicotte V. Ivers H. Savard J. (2021). COVID-19 pandemic stressors and psychological symptoms in breast cancer patients. Curr. Oncol. 28, 294–300. doi: 10.3390/curroncol28010034, 33430131 PMC7903274

[ref24] McEwen B. S. (1998). Protective and damaging effects of stress mediators. N. Engl. J. Med. 338, 171–179. doi: 10.1056/NEJM199801153380307, 9428819

[ref25] Nanni M. G. Caruso R. Travado L. Ventura C. Palma A. Berardi A. M. . (2018). Relationship of demoralization with anxiety, depression, and quality of life: a southern European study of Italian and Portuguese cancer patients. Psychooncology. 27, 2616–2622. doi: 10.1002/pon.4824, 29943491

[ref26] Nikoloudi M. Galanis P. Tsatsou I. Mystakidou K. (2025). Demoralization, pain, and hope as predictors of desire for death in cancer patients: a cross-sectional study. J. Pain Symptom Manag. 70, 295–301. doi: 10.1016/j.jpainsymman.2025.05.018, 40482786

[ref9003] Palgi Y. Greenblatt-Kimron L. Hoffman Y. Segel-Karpas D. Ben-David B. Shenkman G. . (2024). PTSD symptoms and subjective traumatic outlook in the Israel-Hamas war: Capturing a broader picture of posttraumatic reactions. Psychiatry Res. 339, 116096. doi: 10.1016/j.psychres.2024.116096 39067235

[ref27] Ramm M. Schnabel K. Jedamzik J. Jürgens L. Rassenhofer M. Brähler E. . (2025). Demoralization's link to depression and anxiety symptoms: a network analysis. J. Affect. Disord. 372, 491–501. doi: 10.1016/j.jad.2024.12.045, 39675678

[ref28] Raut S. (2025). Dual fronts: the psychological and logistical burden of war on oncology patients. JCO Glob. Oncol. 11:e2500256. doi: 10.1200/GO-25-00256, 41092266

[ref29] Robinson S. Kissane D. W. Brooker J. Burney S. Clarke D. M. (2016a). Refinement and revalidation of the demoralization scale: the DS-II—internal validity. Cancer 122, 2251–2259. doi: 10.1002/cncr.29921, 27171617

[ref30] Robinson S. Kissane D. W. Brooker J. Burney S. Clarke D. M. (2016b). Refinement and revalidation of the demoralization scale: the DS-II—external validity. Cancer 122, 2260–2267. doi: 10.1002/cncr.29922 27171544

[ref31] Rodin G. Lo C. Mikulincer M. Donner A. Gagliese L. Zimmermann C. (2009). Pathways to distress: the multiple determinants of depression, hopelessness, and the desire for hastened death in metastatic cancer patients. Soc. Sci. Med. 68, 562–569. doi: 10.1016/j.socscimed.2008.10.037, 19059687

[ref32] Sender A. Hinz A. Broemer L. Mehnert-Theuerkauf A. Strauß B. Rosendahl J. (2025). Demoralization in breast cancer survivors. Front. Psychol. 16:1523164. doi: 10.3389/fpsyg.2025.1523164, 40718556 PMC12292496

[ref33] Sowan W. Baziliansky S. (2024). The moderating role of demoralization on the association between exposure to war and posttraumatic stress symptoms among Israeli civilians in reaction to the October 7 war: a longitudinal study. Clin. Psychol. Psychother. 31:e70021. doi: 10.1002/cpp.70021, 39623758 PMC11612540

[ref34] Sowan W. Kissane D. (2024). Demoralization and well-being among self-employed individuals with cardiac disease: the role of intolerance of uncertainty. Front. Psychol. 15:1388032. doi: 10.3389/fpsyg.2024.1388032, 39021650 PMC11253239

[ref35] Tang W. Z. Cheng S. L. Mangantig E. Hanum P. I. Y. Jia K. Yusuf A. (2025). Factors correlated with demoralization among cancer patients: a systematic review and meta-analysis. Palliat. Support. Care 23, 1–11. doi: 10.1017/S1478951524001597 PMC13168777 39831587

[ref36] Tecuta L. Tomba E. Grandi S. Fava G. A. (2015). Demoralization: a systematic review on its clinical characterization. Psychol. Med. 45, 673–691. doi: 10.1017/S0033291714001597, 25032712

[ref37] Vehling S. Kissane D. W. Lo C. Glaesmer H. Hartung T. J. Rodin G. . (2017). The association of demoralization with mental disorders and suicidal ideation in patients with cancer. Cancer 123, 3394–3401. doi: 10.1002/cncr.30749, 28472548

[ref38] Vehling S. Oechsle K. Koch U. Mehnert A. (2013). Receiving palliative treatment moderates the effect of age and gender on demoralization in patients with cancer. PLoS One 8:e59417. doi: 10.1371/journal.pone.0059417, 23555030 PMC3598752

[ref39] Vesco P. Baliki G. Brück T. Döring S. Eriksson A. Fjelde H. . (2025). The impacts of armed conflict on human development: a review of the literature. World Dev. 187:106806. doi: 10.1016/j.worlddev.2024.106806

[ref40] Wang Y. Sun H. Ji Q. Wu Q. Wei J. Zhu P. (2023). Prevalence, associated factors and adverse outcomes of demoralization in cancer patients: a decade of systematic review. Am. J. Hosp. Palliat. Care 40, 1216–1230. doi: 10.1177/10499091231154887, 36718669

[ref41] Wilson E. (2019). Social work, cancer survivorship and liminality: meeting the needs of young women diagnosed with early stage breast cancer. J. Soc. Work. Pract. 34, 95–111. doi: 10.1080/02650533.2019.1604497

[ref42] Wu Y.-C. Tung H.-H. Wei J. (2019). Quality of life, demoralization syndrome and health-related lifestyle in cardiac transplant recipients: a longitudinal study in Taiwan. Eur. J. Cardiovasc. Nurs. 18, 149–162. doi: 10.1177/1474515118800397, 30226074

[ref43] Xiong Y. Li H. Cai K. Yu M. Zhou J. Li J. . (2025). Demoralization and dignity loss in breast cancer: a network analysis and computer simulation study. Asia-Pac. J. Oncol. Nurs. 12:100803. doi: 10.1016/j.apjon.2025.100803, 41210322 PMC12595355

[ref9004] Yahav R. Cohen M. (2007). Symptoms of acute stress in Jewish and Arab Israeli citizens during the Second Lebanon War. Soc. Psychiatry Psychiatr. Epidemiol. 42, 830–836. doi: 10.1007/s00127-007-0237-5 17668139

